# Author Correction: Rare genetic variants affecting urine metabolite levels link population variation to inborn errors of metabolism

**DOI:** 10.1038/s41467-021-26242-7

**Published:** 2021-10-06

**Authors:** Yurong Cheng, Pascal Schlosser, Johannes Hertel, Peggy Sekula, Peter J. Oefner, Ute Spiekerkoetter, Johanna Mielke, Daniel F. Freitag, Miriam Schmidts, GCKD Investigators, Peter J. Oefner, Florian Kronenberg, Kai-Uwe Eckardt, Florian Kronenberg, Kai-Uwe Eckardt, Ines Thiele, Yong Li, Anna Köttgen

**Affiliations:** 1 Institute of Genetic Epidemiology, Faculty of Medicine and Medical Center, University of Freiburg, Freiburg, Germany; 2 Faculty of Biology, University of Freiburg, Freiburg, Germany; 3 School of Medicine, National University of Ireland, Galway, Galway, Ireland; 4 Department of Psychiatry and Psychotherapy, University Medicine Greifswald, University of Greifswald, Greifswald, Germany; 5 Institute of Functional Genomics, University of Regensburg, Regensburg, Germany; 6 Department of General Pediatrics and Adolescent Medicine, Medical Center and Faculty of Medicine, University of Freiburg, Freiburg, Germany; 7 Division Pharmaceuticals, Open Innovation & Digital Technologies, Bayer AG, Wuppertal, Germany; 8 Department of Nephrology and Hypertension, University of Erlangen-Nürnberg, Erlangen, Germany; 9 Institute of Genetic Epidemiology, Department of Genetics and Pharmacology, Medical University of Innsbruck, Innsbruck, Austria; 10 Department of Nephrology and Medical Intensive Care, Charité—Universitätsmedizin Berlin, Berlin, Germany; 11 Division of Microbiology, National University of Ireland, Galway, Galway, Ireland; 12 APC Microbiome Ireland, Galway, Ireland; 13 CIBSS—Centre for Integrative Biological Signalling Studies, Albert-Ludwigs-Universität Freiburg, Freiburg, Germany

**Keywords:** Computational models, Genome-wide association studies, Metabolic disorders

Correction to: *Nature Communications* 10.1038/s41467-020-20877-8, published online 11 February 2021.

The original version of the Supplementary Data associated with this Article included two incorrect column labels in Supplementary Data 3 and 6 files. All numeric content remains unchanged. The HTML has been updated to include a corrected version of Supplementary Data 3 and 6; the original version of Supplementary Data 3 and 6 with the incorrectly labelled column headers can be found as Supplementary Information associated with this Correction.

## Supplementary information

Supplementary Information

