# Supplementary material for: Rare genetic variants affecting urine metabolite levels link population variation to inborn errors of metabolism
Source: Nat Commun. 2021 Feb 11;12:964. doi: 10.1038/s41467-020-20877-8 (PMC7878905; doi:10.1038/s41467-020-20877-8)
Supplement: Supplementary file 1 — Supplementary Information [file 41467_2020_20877_MOESM1_ESM.pdf]

# Supplementary Information

## Rare genetic variants affecting urine metabolite levels link population variation to inborn errors of metabolism

Yurong Cheng, Pascal Schlosser, Johannes Hertel, Peggy Sekula, Peter J. Oefner, Ute Spiekerkoetter, Johanna Mielke, Daniel F. Freitag, Miriam Schmidts, GCKD Investigators, Florian Kronenberg, Kai-Uwe Eckardt, Ines Thiele, Yong Li, Anna Köttgen

**Supplementary Figure 1:** Metabolite levels by qualifying variant carrier status of significant genes ......... 2

**Supplementary Figure 2:** Expression of metabolite-associated genes in murine kidney cell types. ......... 17

**Supplementary Figure 3:** Expression of metabolite-associated genes in human kidney cell types. .......... 18

**Supplementary Note 1:** Extended acknowledgements ........................................................................ 19

**Supplementary Note 2:** Detailed information about non-targeted mass spectrometry analysis and the identification of metabolites .................................................................................................................. 20

**Supplementary Figure 1:** Metabolite levels by qualifying variant carrier status of significant genes

The Y axis represents metabolite levels after inverse normal transformation (INV), which allows for comparisons across metabolites. Units correspond to standard deviations. The symbol color indicates observed rare variant carrier status (gray for individuals who do not carry any qualifying variants included in the gene-based tests, blue for heterozygous carriers, orange for homozygous carriers, and mulberry for carriers of multiple variants in the heterozygous state). Symbol shape indicates variant consequence (round for nonsynonymous, square for splice, triangle for stop). Box plots show the distribution of metabolite levels by rare variant carrier status for each of the 53 significant gene-metabolite pairs. The box ranges from the 25$^{th}$ to 75$^{th}$ percentile of transformed metabolite levels, the median is indicated by a line, and whiskers end at the last observed value within 1.5*(interquartile range) away from the box.

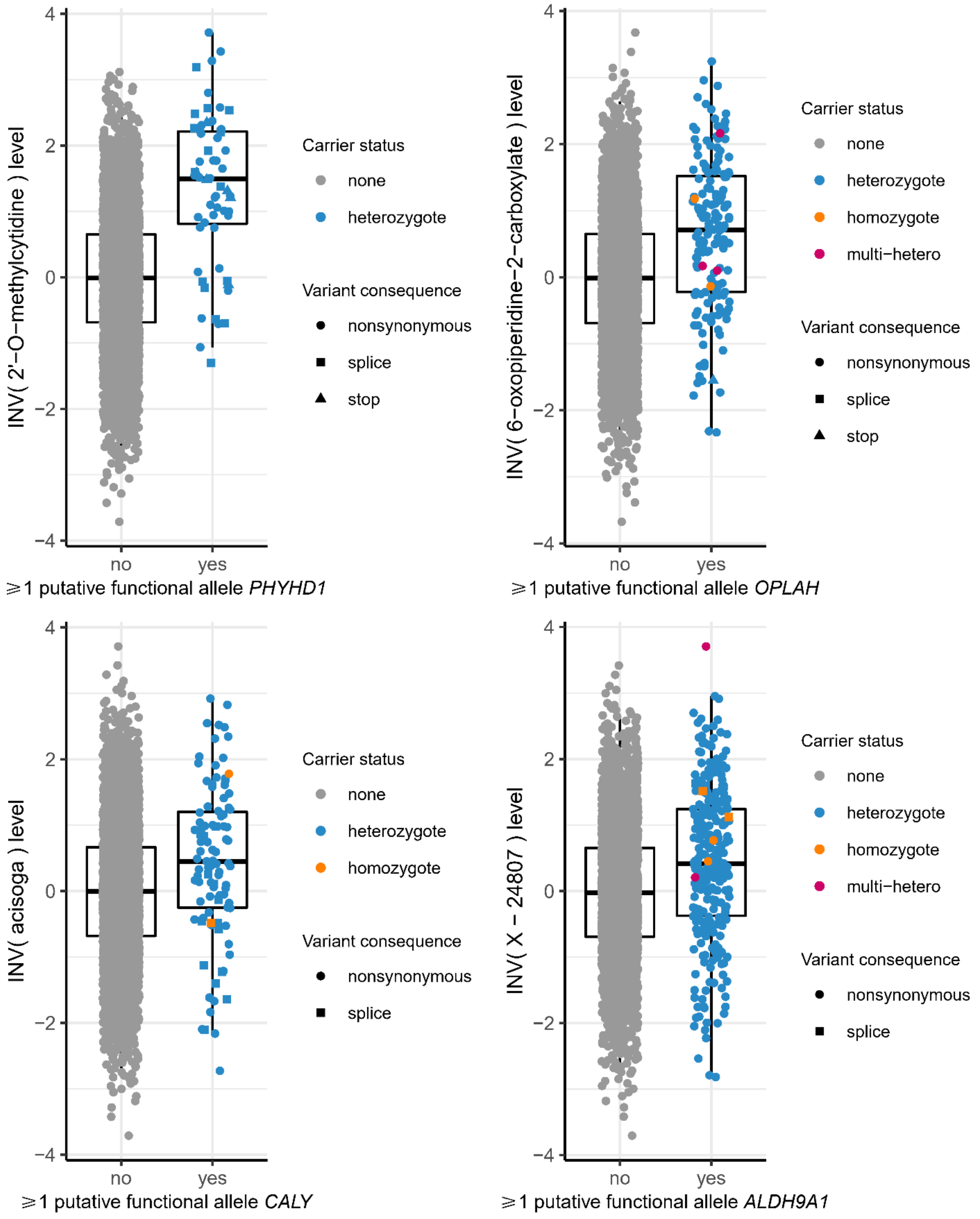
INV( 2'−O−methylcytidine ) level
4
2
0
−2
−4
no
yes
⩾1 putative functional allele PHYHD1
Carrier status
none
heterozygote
Variant consequence
nonsynonymous
splice
stop
INV( 6−oxopiperidine−2−carboxylate ) level
⩾1 putative functional allele OPLAH
Carrier status
none
heterozygote
homozygote
multi−hetero
Variant consequence
nonsynonymous
splice
stop
INV( acisoga ) level
⩾1 putative functional allele CALY
Carrier status
none
heterozygote
homozygote
Variant consequence
nonsynonymous
splice
INV( X − 24807 ) level
⩾1 putative functional allele ALDH9A1
Carrier status
none
heterozygote
homozygote
multi−hetero
Variant consequence
nonsynonymous
splice

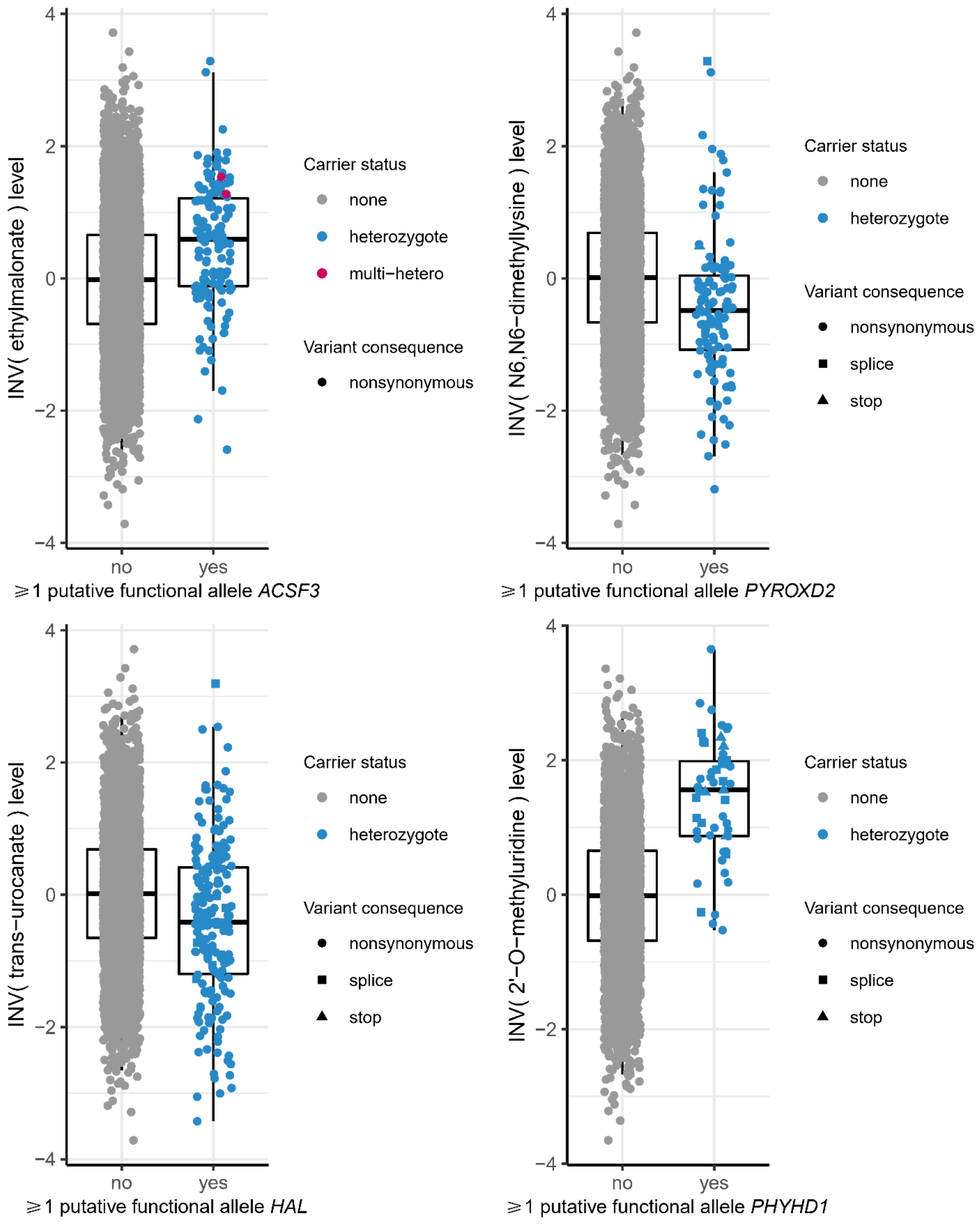
INV( ethylmalonate ) level
no
yes
≥1 putative functional allele ACSF3
Carrier status
none
heterozygote
multi−hetero
Variant consequence
nonsynonymous
INV( N6,N6−dimethyllysine ) level
≥1 putative functional allele PYROXD2
splice
stop
INV( trans−urocanate ) level
≥1 putative functional allele HAL
INV( 2'−O−methyluridine ) level
≥1 putative functional allele PHYHD1

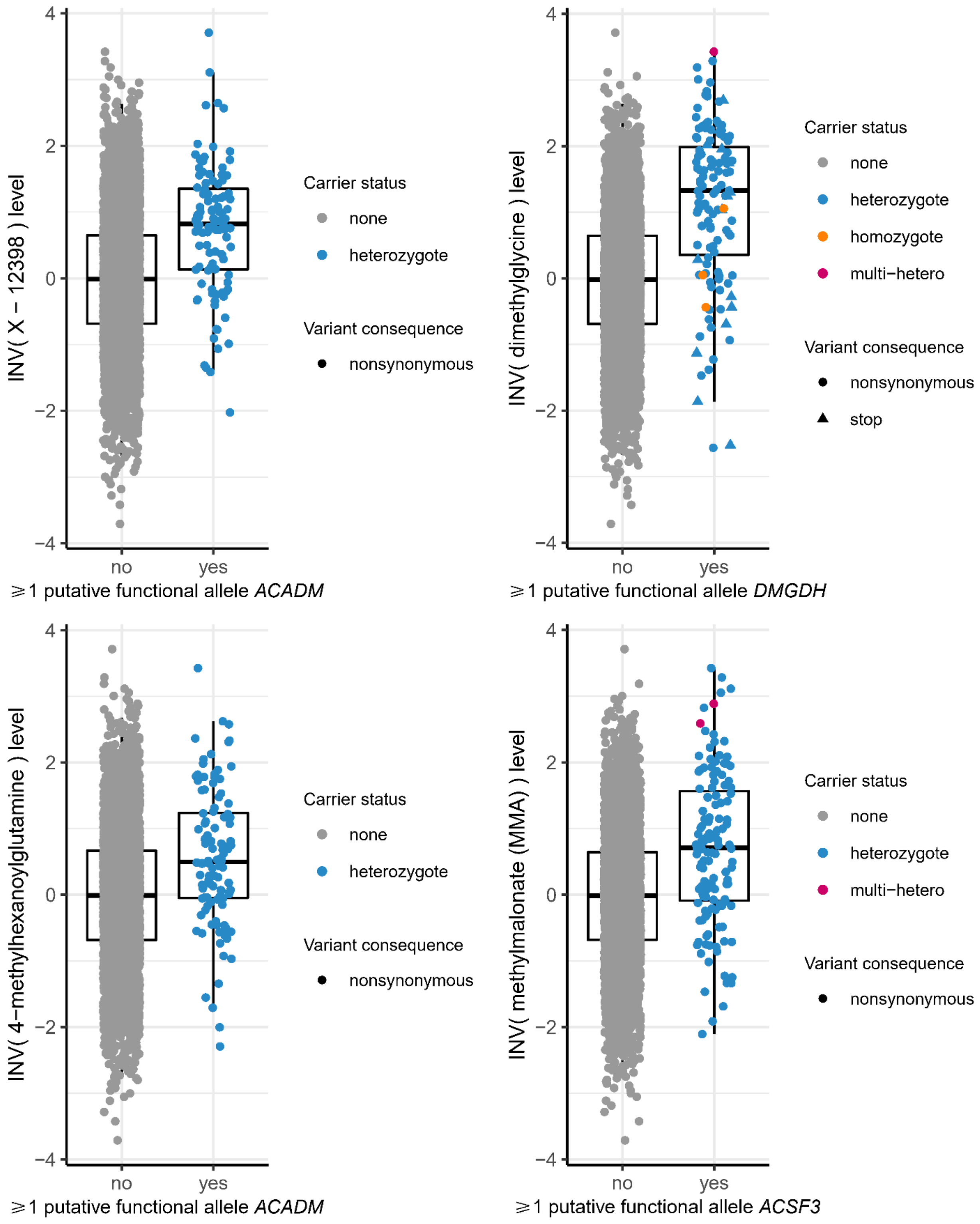

INV( X − 12398 ) level
≥1 putative functional allele *ACADM*
no
yes
Carrier status
none
heterozygote
Variant consequence
nonsynonymous
INV( dimethylglycine ) level
≥1 putative functional allele *DMGDH*
Carrier status
none
heterozygote
homozygote
multi−hetero
Variant consequence
nonsynonymous
stop
INV( 4−methylhexanoylglutamine ) level
≥1 putative functional allele *ACADM*
Carrier status
none
heterozygote
Variant consequence
nonsynonymous
INV( methylmalonate (MMA) ) level
≥1 putative functional allele *ACSF3*
Carrier status
none
heterozygote
multi−hetero
Variant consequence
nonsynonymous

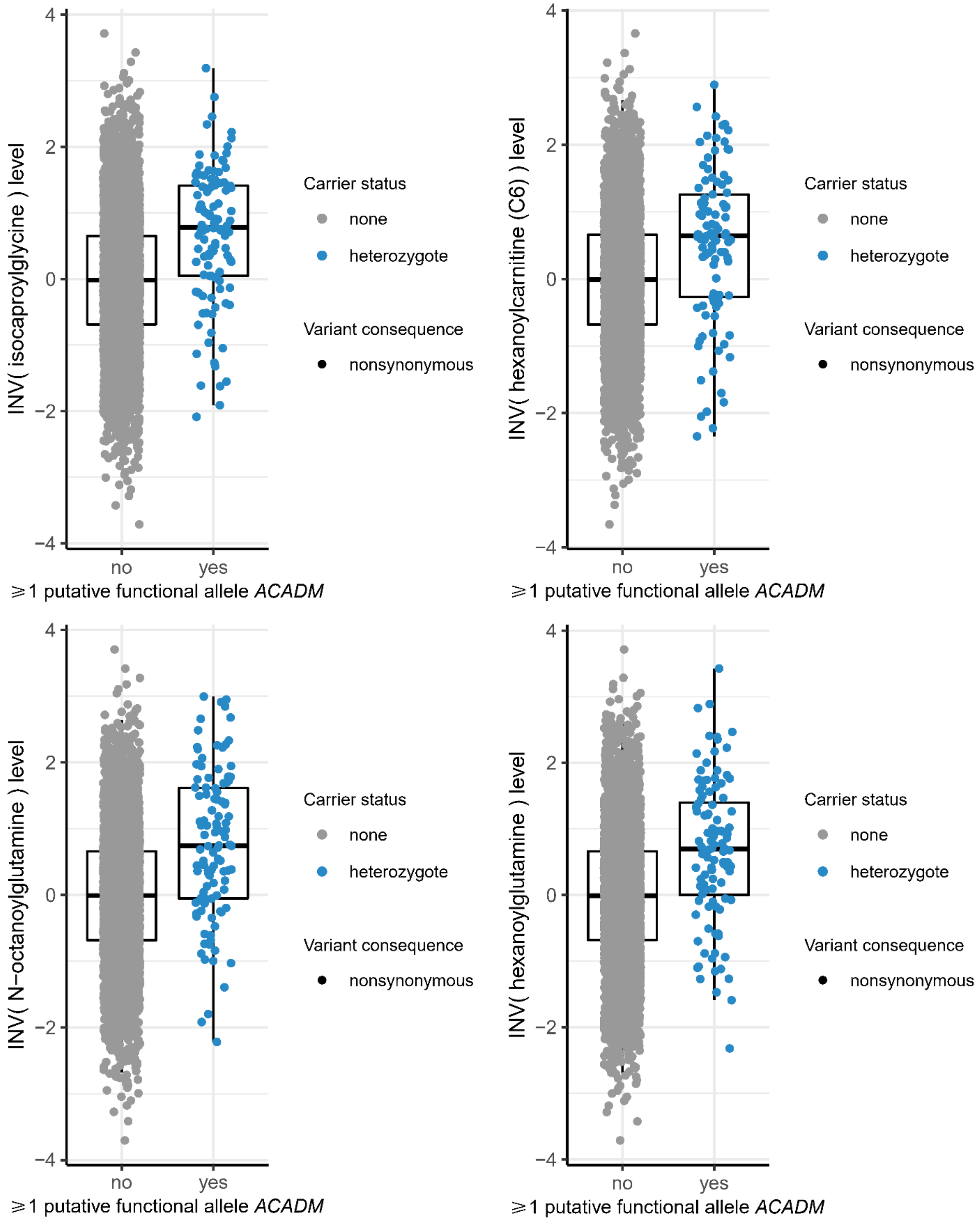

INV( isocaproylglycine ) level
4
2
0
−2
−4
no
yes
⩾1 putative functional allele *ACADM*
Carrier status
none
heterozygote
Variant consequence
nonsynonymous
INV( hexanoylcarnitine (C6) ) level
4
2
0
−2
−4
no
yes
⩾1 putative functional allele *ACADM*
Carrier status
none
heterozygote
Variant consequence
nonsynonymous
INV( N−octanoylglutamine ) level
4
2
0
−2
−4
no
yes
⩾1 putative functional allele *ACADM*
Carrier status
none
heterozygote
Variant consequence
nonsynonymous
INV( hexanoylglutamine ) level
4
2
0
−2
−4
no
yes
⩾1 putative functional allele *ACADM*
Carrier status
none
heterozygote
Variant consequence
nonsynonymous

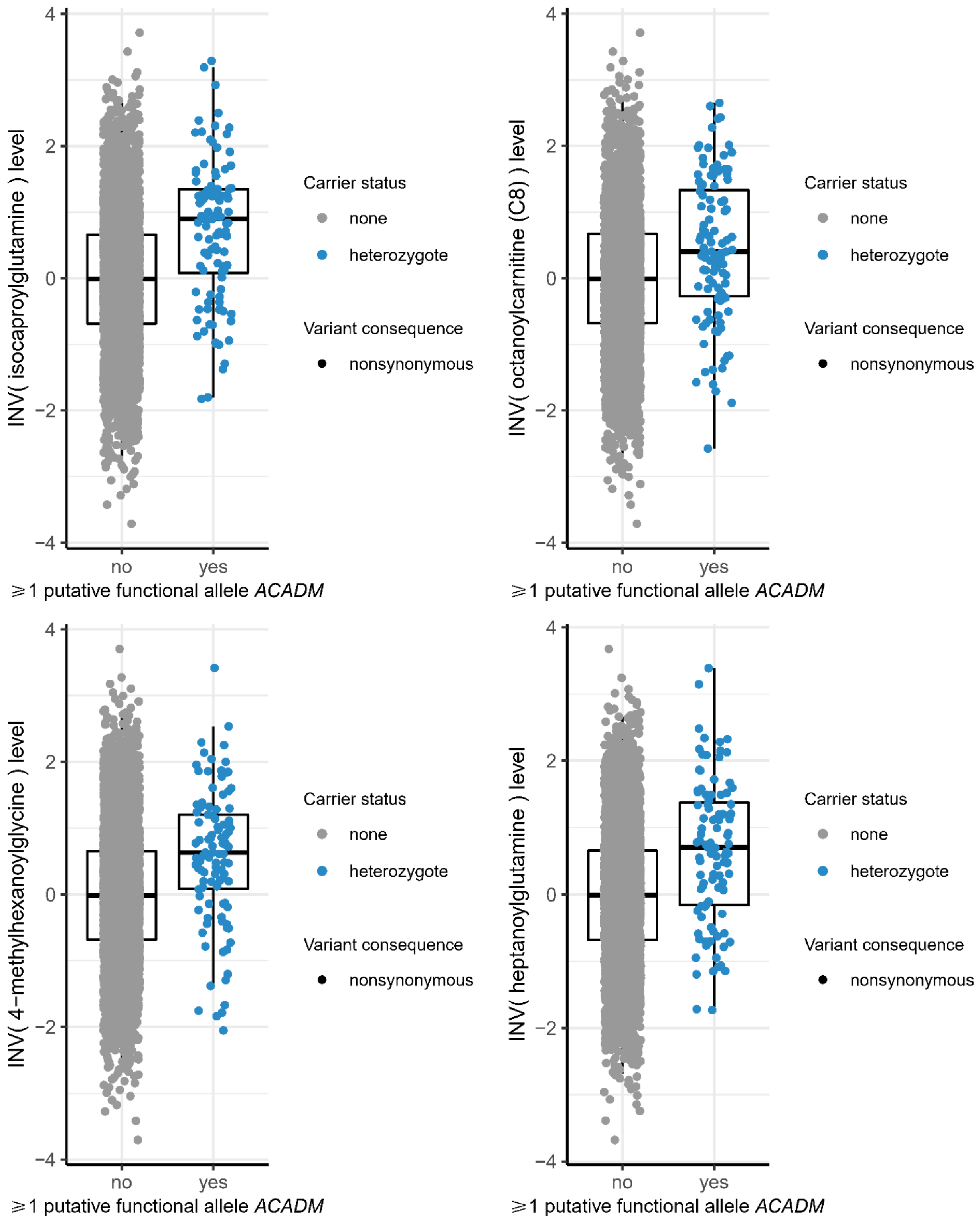
INV( isocaproylglutamine ) level
INV( octanoylcarnitine (C8) ) level
INV( 4−methylhexanoylglycine ) level
INV( heptanoylglutamine ) level
4
2
0
−2
−4
no
yes
≥1 putative functional allele *ACADM*
Carrier status
none
heterozygote
Variant consequence
nonsynonymous

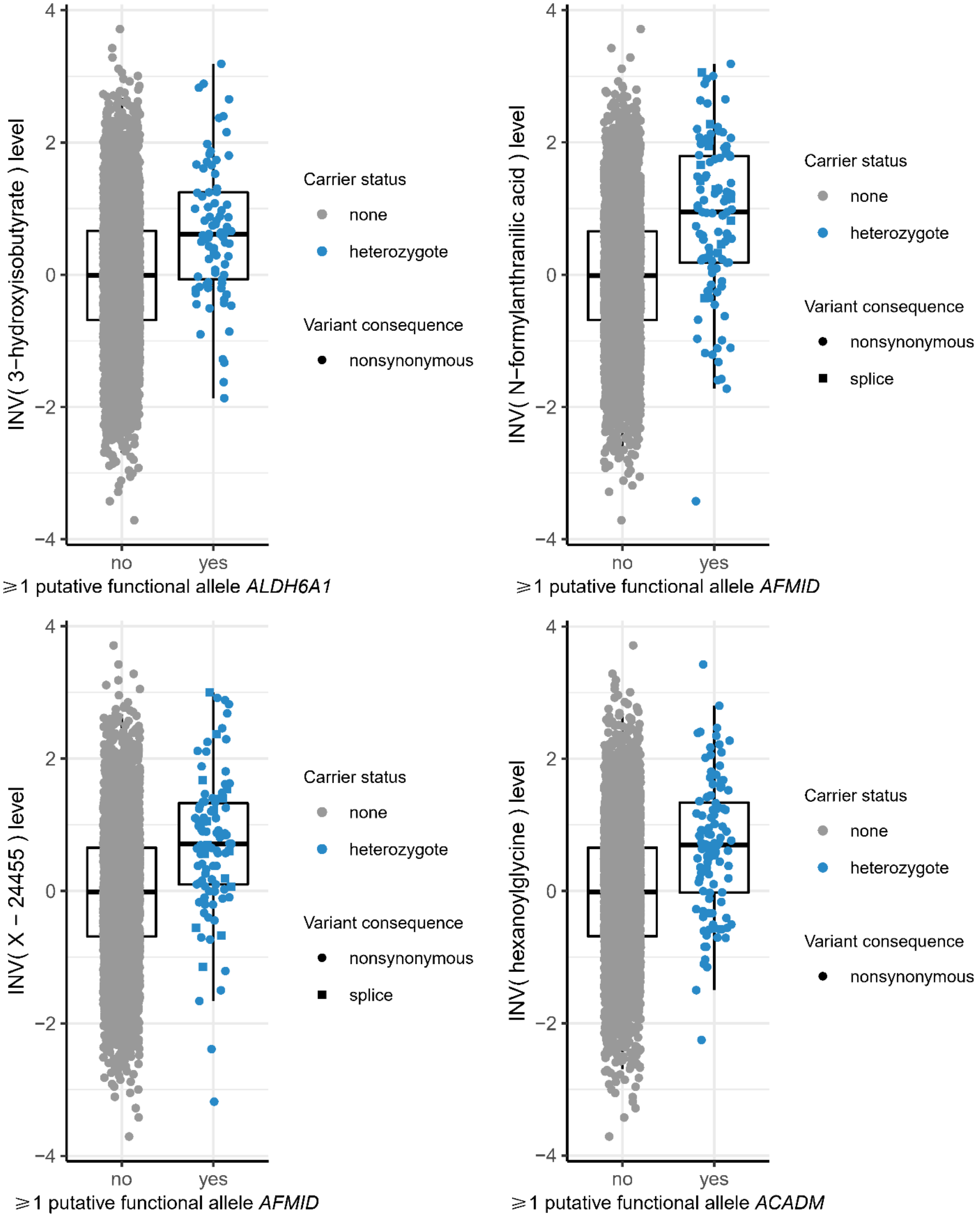
INV( 3−hydroxyisobutyrate ) level
4
2
0
−2
−4
no
yes
⩾1 putative functional allele ALDH6A1
Carrier status
none
heterozygote
Variant consequence
nonsynonymous
INV( N−formylanthranilic acid ) level
4
2
0
−2
−4
no
yes
⩾1 putative functional allele AFMID
Carrier status
none
heterozygote
Variant consequence
nonsynonymous
splice
INV( X − 24455 ) level
4
2
0
−2
−4
no
yes
⩾1 putative functional allele AFMID
Carrier status
none
heterozygote
Variant consequence
nonsynonymous
splice
INV( hexanoylglycine ) level
4
2
0
−2
−4
no
yes
⩾1 putative functional allele ACADM
Carrier status
none
heterozygote
Variant consequence
nonsynonymous

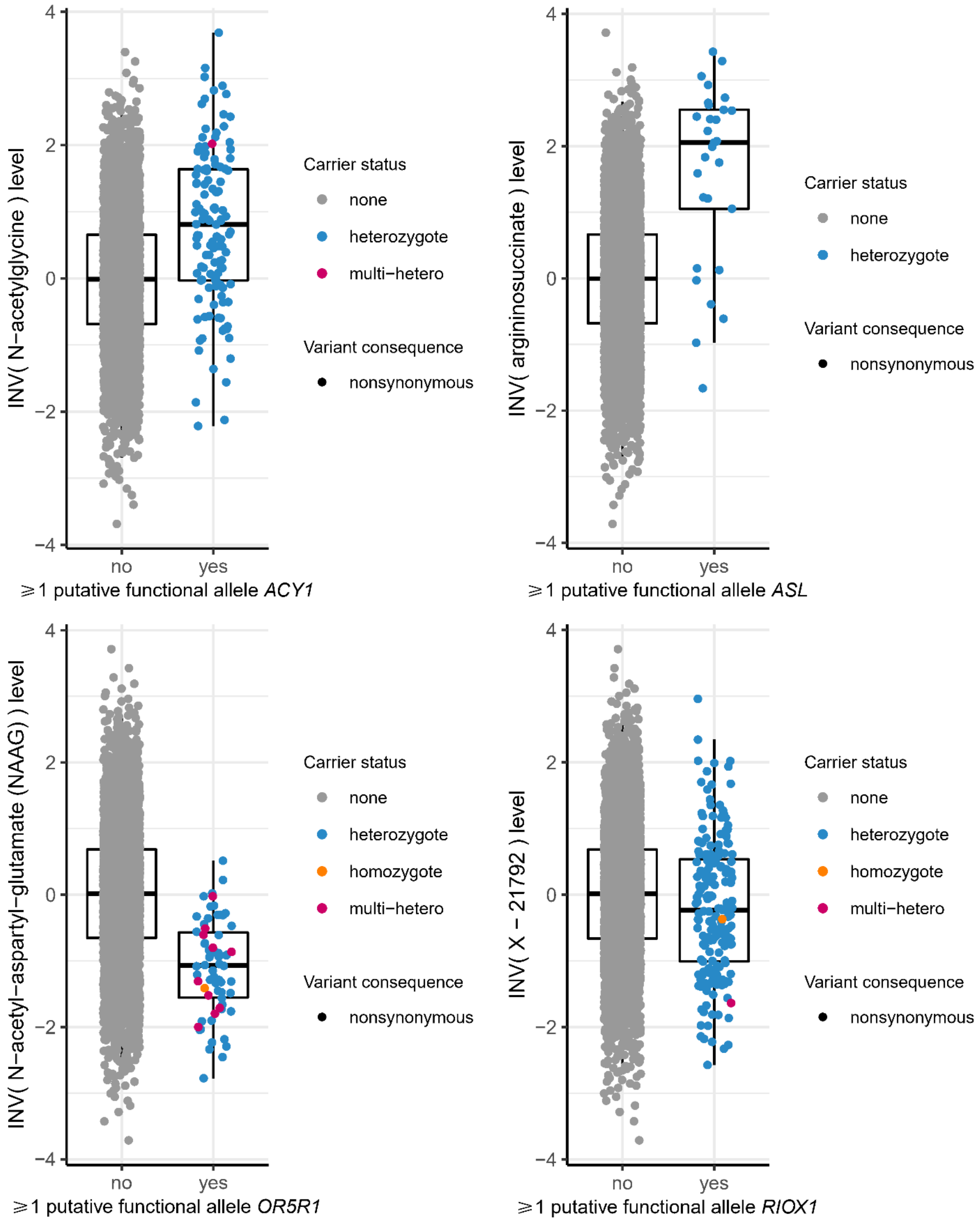

INV( N−acetylglycine ) level
4
2
0
−2
−4
no
yes
≥1 putative functional allele *ACY1*
Carrier status
none
heterozygote
multi−hetero
Variant consequence
nonsynonymous
INV( argininosuccinate ) level
4
2
0
−2
−4
no
yes
≥1 putative functional allele *ASL*
Carrier status
none
heterozygote
Variant consequence
nonsynonymous
INV( N−acetyl−aspartyl−glutamate (NAAG) ) level
4
2
0
−2
−4
no
yes
≥1 putative functional allele *OR5R1*
Carrier status
none
heterozygote
homozygote
multi−hetero
Variant consequence
nonsynonymous
INV( X − 21792 ) level
4
2
0
−2
−4
no
yes
≥1 putative functional allele *RIOX1*
Carrier status
none
heterozygote
homozygote
multi−hetero
Variant consequence
nonsynonymous

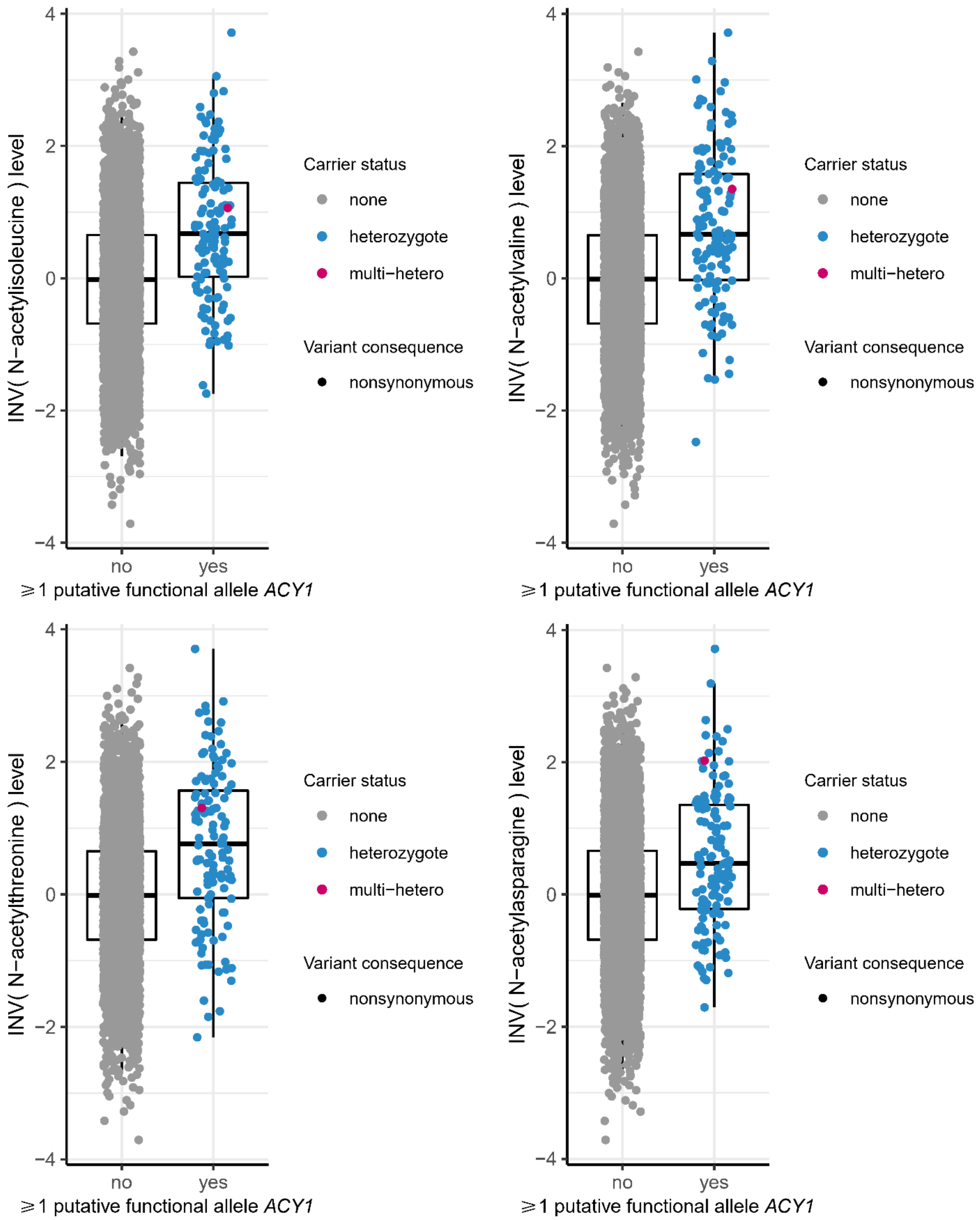
INV( N−acetylisoleucine ) level
INV( N−acetylvaline ) level
INV( N−acetylthreonine ) level
INV( N−acetylasparagine ) level
4
2
0
−2
−4
no
yes
⩾1 putative functional allele *ACY1*
Carrier status
none
heterozygote
multi−hetero
Variant consequence
nonsynonymous

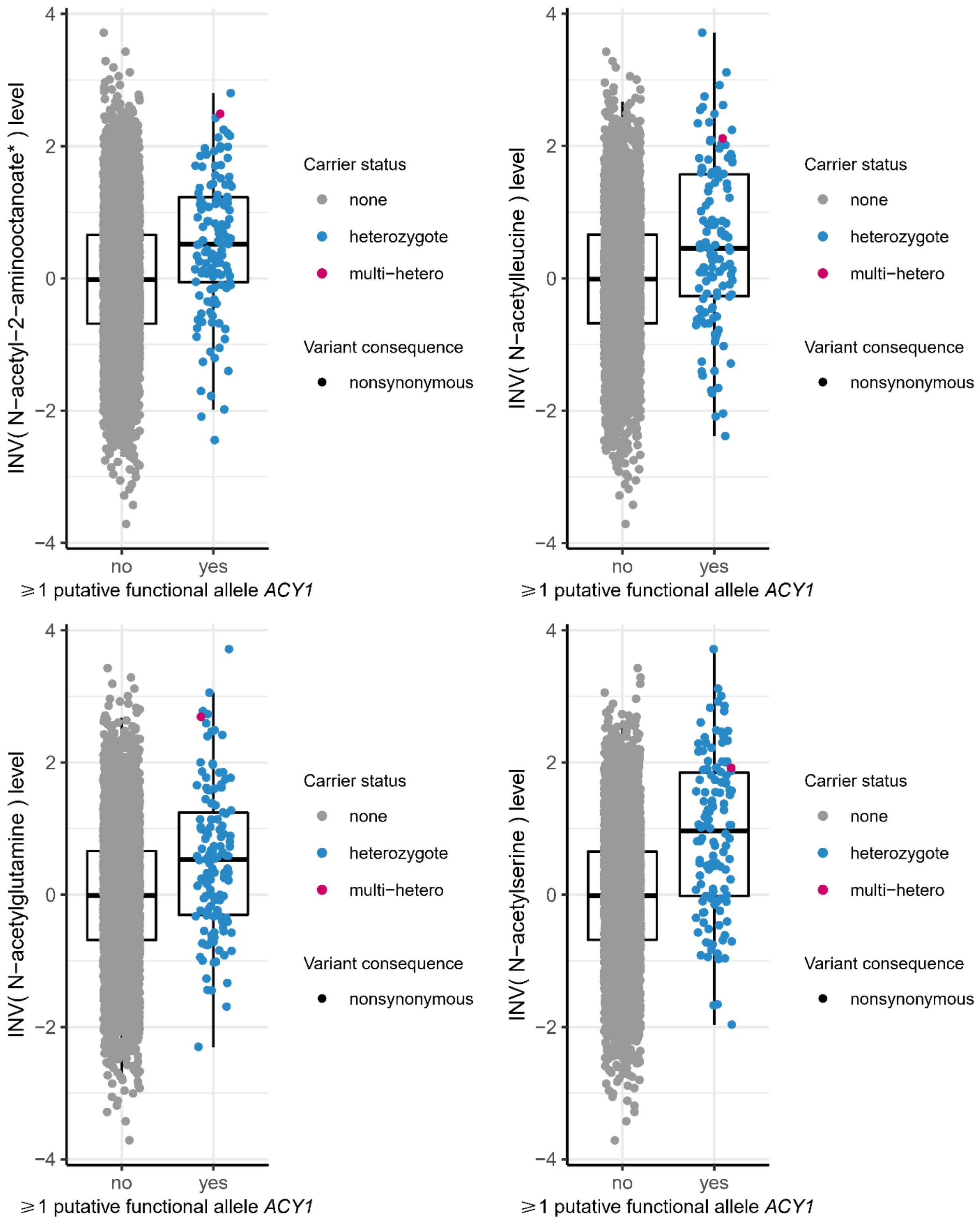

INV( N−acetyl−2−aminooctanoate* ) level
INV( N−acetylleucine ) level
INV( N−acetylglutamine ) level
INV( N−acetylserine ) level
4
2
0
−2
−4
no
yes
⩾1 putative functional allele *ACY1*
Carrier status
none
heterozygote
multi−hetero
Variant consequence
nonsynonymous

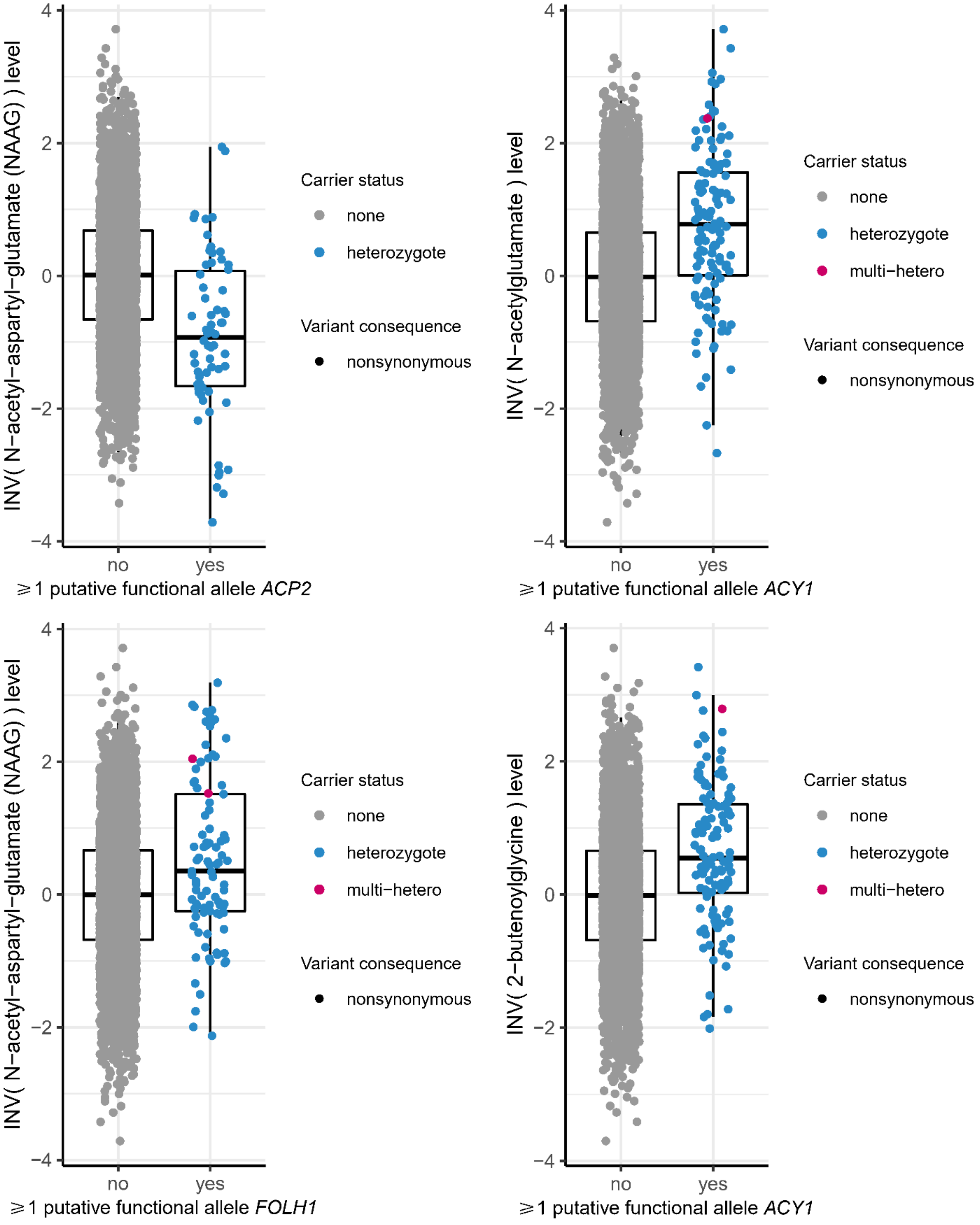
INV( N−acetyl−aspartyl−glutamate (NAAG) ) level
4
2
0
−2
−4
no
yes
≥1 putative functional allele *ACP2*
Carrier status
none
heterozygote
Variant consequence
nonsynonymous
INV( N−acetylglutamate ) level
≥1 putative functional allele *ACY1*
Carrier status
none
heterozygote
multi−hetero
Variant consequence
nonsynonymous
INV( N−acetyl−aspartyl−glutamate (NAAG) ) level
≥1 putative functional allele *FOLH1*
Carrier status
none
heterozygote
multi−hetero
Variant consequence
nonsynonymous
INV( 2−butenoylglycine ) level
≥1 putative functional allele *ACY1*
Carrier status
none
heterozygote
multi−hetero
Variant consequence
nonsynonymous

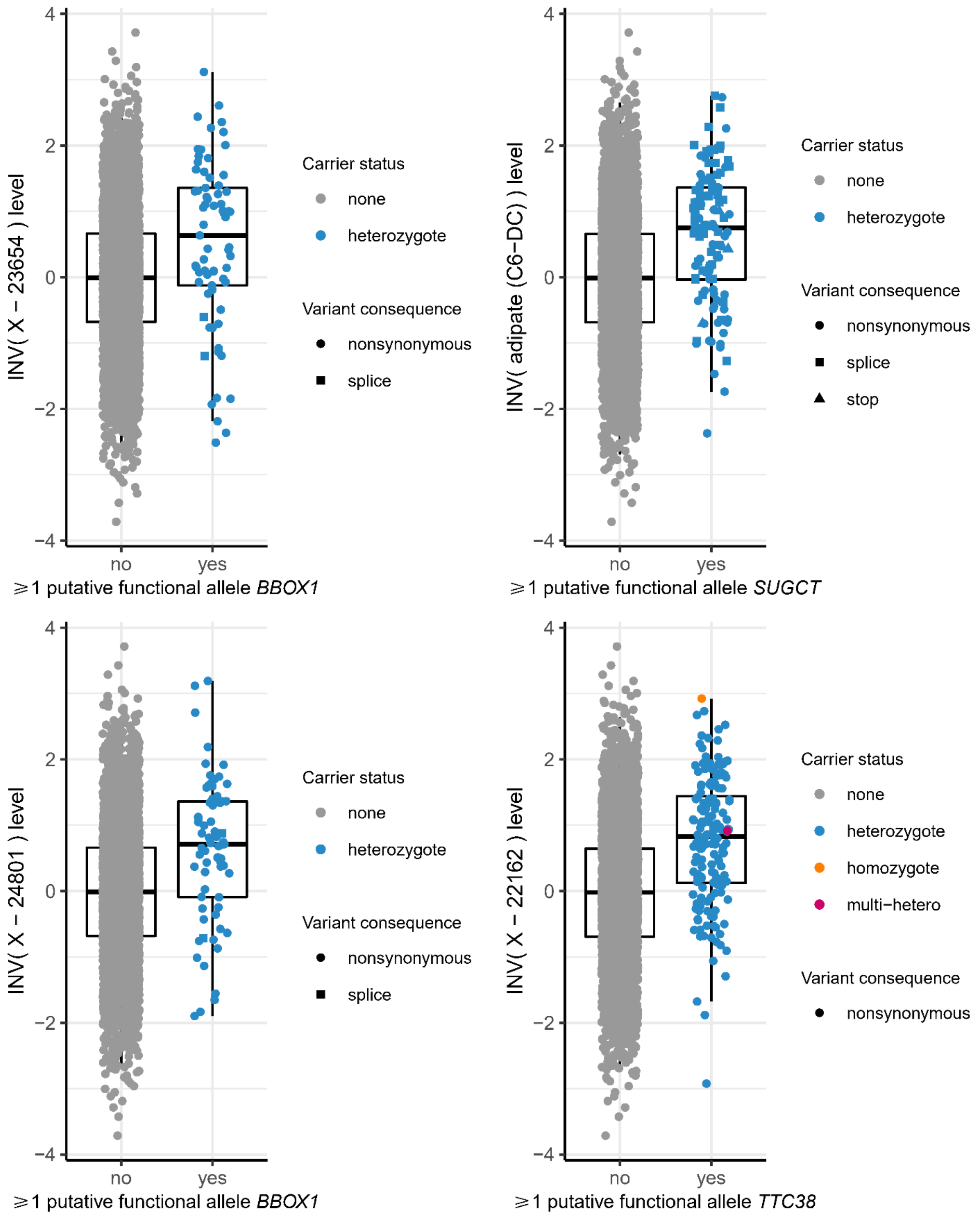
4
2
0
−2
−4
INV( X − 23654 ) level
no
yes
⩾1 putative functional allele BBOX1
Carrier status
none
heterozygote
Variant consequence
nonsynonymous
splice
INV( adipate (C6−DC) ) level
⩾1 putative functional allele SUGCT
stop
INV( X − 24801 ) level
INV( X − 22162 ) level
⩾1 putative functional allele TTC38
homozygote
multi−hetero

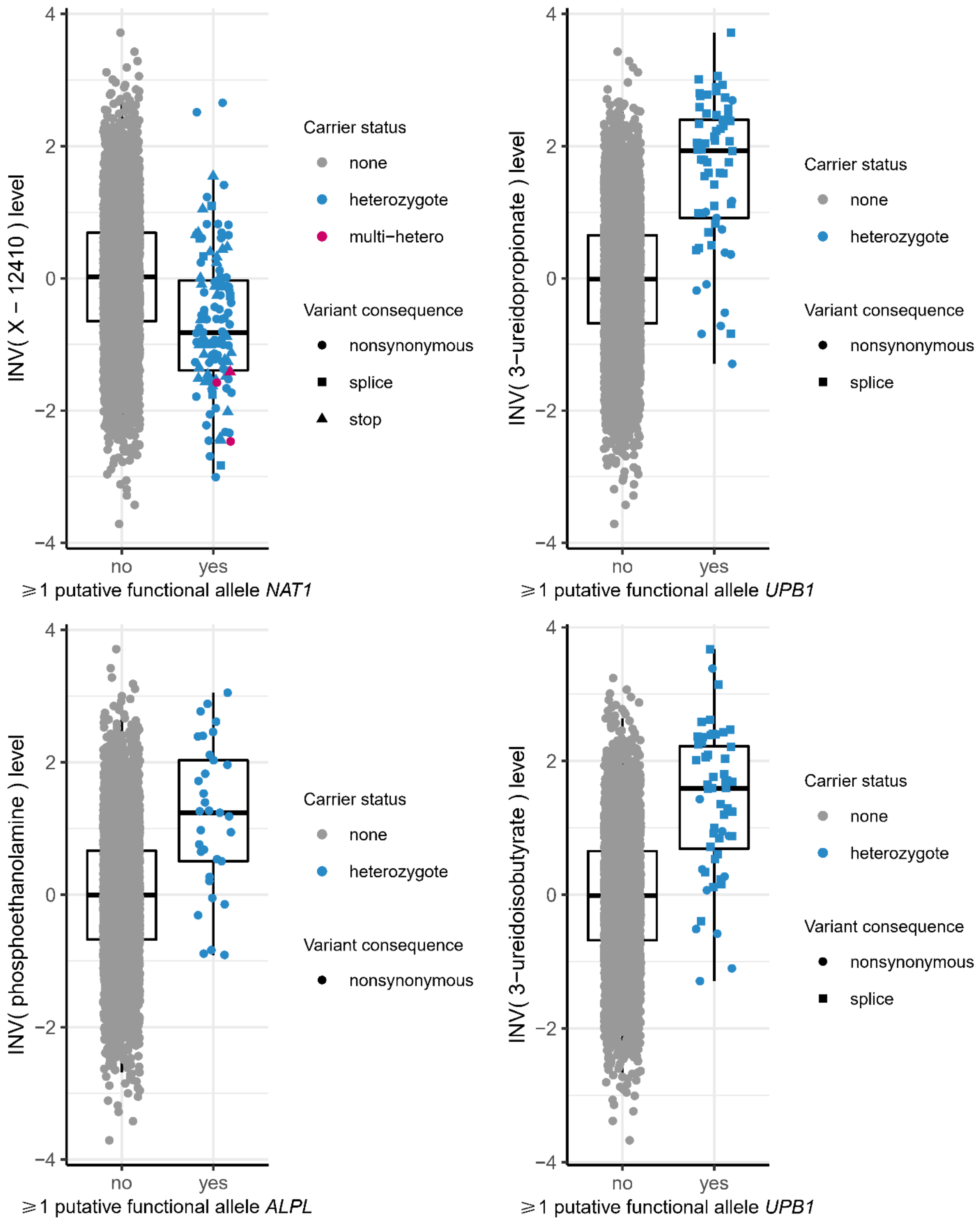
INV( X − 12410 ) level
≥1 putative functional allele *NAT1*
no
yes
Carrier status
none
heterozygote
multi−hetero
Variant consequence
nonsynonymous
splice
stop
INV( 3−ureidopropionate ) level
≥1 putative functional allele *UPB1*
Carrier status
none
heterozygote
Variant consequence
nonsynonymous
splice
INV( phosphoethanolamine ) level
≥1 putative functional allele *ALPL*
Carrier status
none
heterozygote
Variant consequence
nonsynonymous
INV( 3−ureidoisobutyrate ) level
≥1 putative functional allele *UPB1*
Carrier status
none
heterozygote
Variant consequence
nonsynonymous
splice

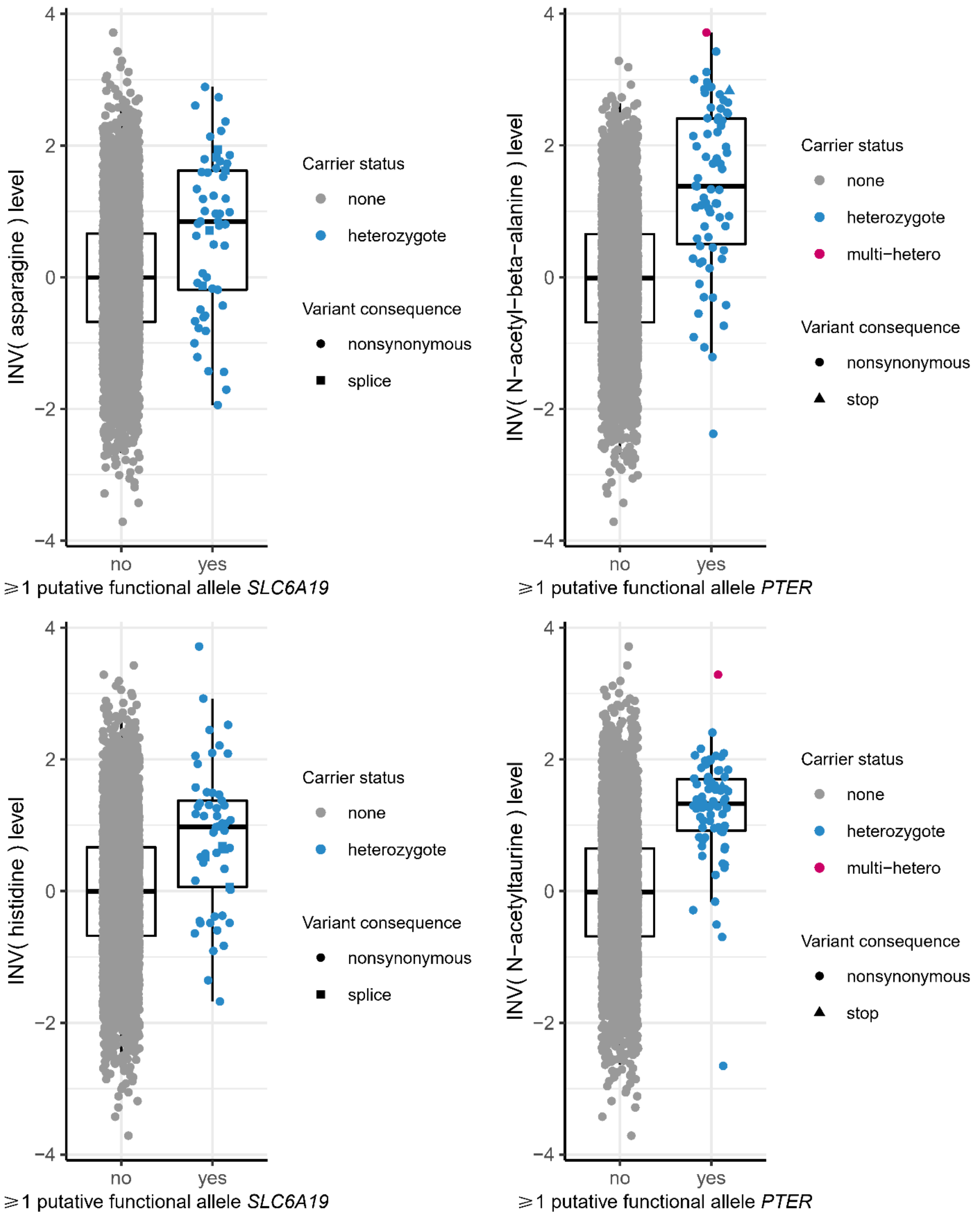
INV( asparagine ) level
4
2
0
−2
−4
no
yes
⩾1 putative functional allele SLC6A19
Carrier status
none
heterozygote
Variant consequence
nonsynonymous
splice
INV( N−acetyl−beta−alanine ) level
⩾1 putative functional allele PTER
multi−hetero
stop
INV( histidine ) level
INV( N−acetyltaurine ) level

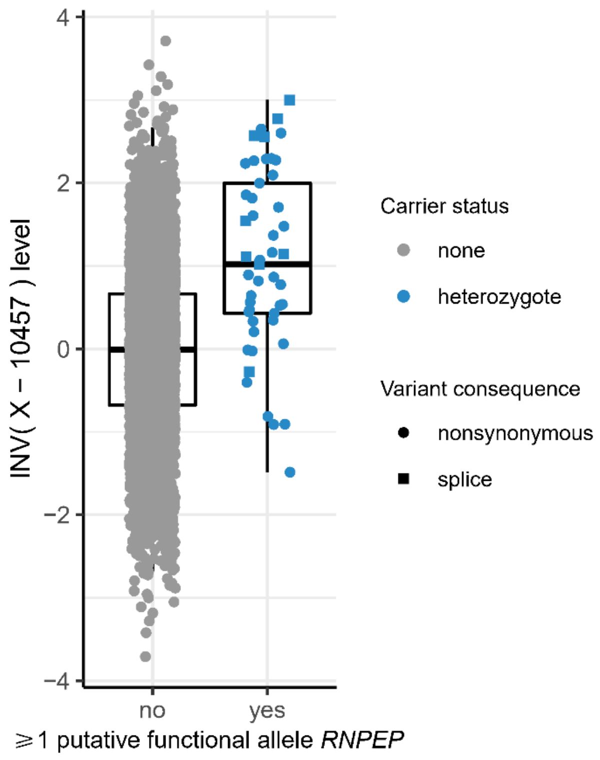
4
2
0
−2
−4
INV( X − 10457 ) level
no
yes
⩾1 putative functional allele *RNPEP*
Carrier status
none
heterozygote
Variant consequence
nonsynonymous
splice

**Supplementary Figure 2:** Expression of metabolite-associated genes in murine kidney cell types.

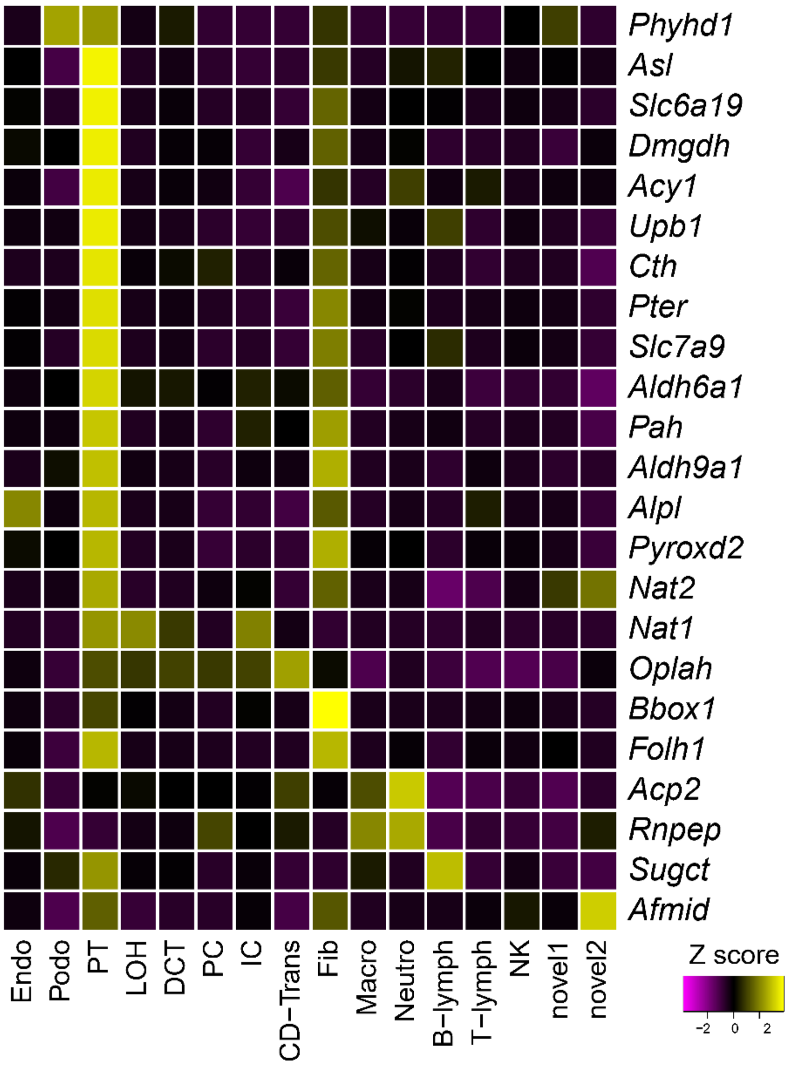


Cell type-specific expression of 30 metabolite-associated genes were based on murine single-cell RNA-sequencing data of Park *et al*. (PMID: 29622724). *Hal* (*HAL*), *Caly* (*CALY*), *Olfr1038-ps* (*OR5R1*) and *Folh1* (*FOLH1*) were not included in this data; 23 genes were highly expressed in at least one cell type. Endo: containing endothelial, vascular, and descending loop of Henle, Podo: podocyte, PT: proximal tubule, LOH: ascending loop of Henle, DCT: distal convoluted tubule, PC: principal cell, IC: intercalated cell, CD-Trans: collecting duct transitional cell, Fib: fibroblast, Macro: macrophage, Neutro: neutrophil, NK: natural killer cell.

**Supplementary Figure 3:** Expression of metabolite-associated genes in human kidney cell types.

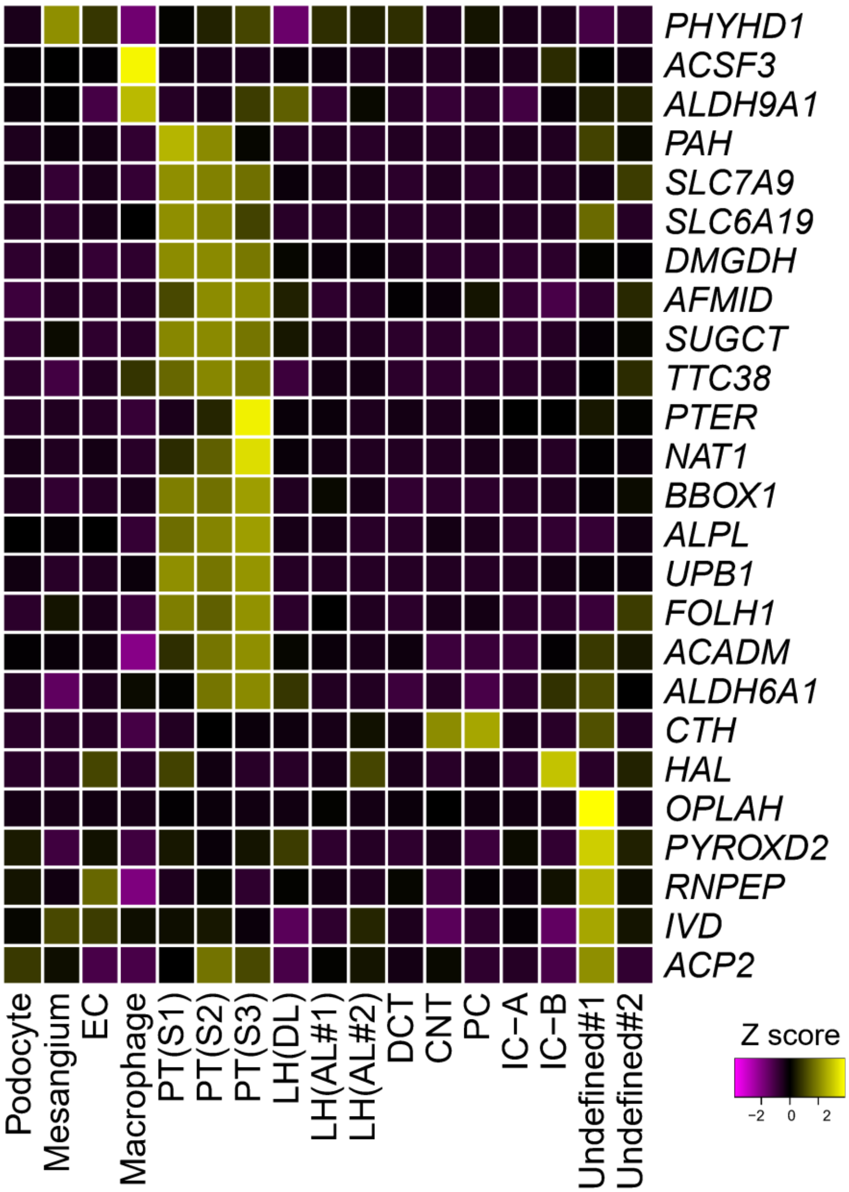


Gene expression levels are based on Wu *et. al*. (PMID: 30449713) single-nucleus RNA-sequencing data. Presented values are mean of z-score transformed expression of cells in each cell type. Only 27 of 30 genes are displayed because *ACY1*, *CALY*, *OR5R1* were not included in Wu's snRNA-seq data. EC: endothelial cells; PT: proximal tubule epithelial cells; LH: loop of Henle; DL: descending limb; AL: ascending limb; DCT: distal convoluted tubule; CNT: connecting segment cells; PC: principal cells; IC: Intercalated cells.

**Supplementary Note 1:** Extended acknowledgements

A list of nephrologists currently collaborating with the GCKD study is available at https://www.gckd.de/intern/sc-gruppe/.

Current GCKD investigators and collaborators with the GCKD study are:

| | |
|---|---|
| University of Erlangen | Kai-Uwe Eckardt, Heike Meiselbach, Markus P. Schneider, Mario Schiffer, Hans-Ulrich Prokosch, Barbara Bärthlein, Andreas Beck, Detlef Kraska, André Reis, Arif B. Ekici, Susanne Becker, Dinah Becker-Grosspitsch, Ulrike Alberth-Schmidt, Birgit Hausknecht, Anke Weigel |
| University of Freiburg | Gerd Walz, Anna Köttgen, Ulla T. Schultheiß, Fruzsina Kotsis, Simone Meder, Erna Mitsch, Ursula Reinhard |
| RWTH Aachen University | Jürgen Floege, Georg Schlieper, Turgay Saritas |
| Charité, University Medicine Berlin | Elke Schaeffner, Seema Baid-Agrawal, Kerstin Theisen |
| Hannover Medical School | Hermann Haller, Jan Menne |
| University of Heidelberg | Martin Zeier, Claudia Sommerer, Rebecca Woitke |
| University of Jena | Gunter Wolf, Martin Busch, Rainer Paul |
| Ludwig-Maximilians University of München | Thomas Sitter |
| University of Würzburg | Christoph Wanner, Vera Krane, Antje Börner-Klein, Britta Bauer |
| Medical University of Innsbruck, Division of Genetic Epidemiology | Florian Kronenberg, Julia Raschenberger, Barbara Kollerits, Lukas Forer, Sebastian Schönherr, Hansi Weissensteiner |
| University of Regensburg, Institute of Functional Genomics | Peter Oefner, Wolfram Gronwald |
| Department of Medical Biometry, Informatics and Epidemiology, University of Bonn | Matthias Schmid, Jennifer Nadal |

**Supplementary Note 2:** Detailed information about non-targeted mass spectrometry analysis and the identification of metabolites

Sample preparation and evaluation of the shipped spot urine samples from participants of the German Chronic Kidney Disease (GCKD) study was performed by Metabolon, Inc. (NC, USA) as described previously[1, 2]. For the purpose of quality control, recovery standards were added before the start of the extraction process. Subsequently, proteins were precipitated with methanol under vigorous shaking for 2 min (Glen Mills Genogrinder 2000) followed by centrifugation to remove protein, to recover chemically diverse metabolites, and to dissociate small, protein-bound molecules or molecules trapped in the precipitated protein matrix. The extract was then separated into several fractions and vacuum dried. Depending on the platform, dried extracts for each sample were dissolved in injection solvent that contained ≥8 injection standards at fixed concentrations. One aliquot each per sample was then analyzed by four different ultra-high performance liquid chromatography-tandem mass spectrometry methods (UPLC-MS/MS):

*1) acidic positive ion conditions*, chromatographically optimized for more hydrophilic compounds. The extract was gradient eluted from a C18 column (Waters UPLC BEH C18-2.1x100 mm, 1.7 µm) using water and methanol, containing 0.05% perfluoropentanoic acid (PFPA) and 0.1% formic acid (FA).

*2) acidic positive ion conditions*, chromatographically optimized for more hydrophobic compounds. Here, the extract was gradient eluted from the same C18 column using methanol, acetonitrile, water, 0.05% PFPA and 0.01% FA, and was operated at an overall higher organic content.

*3) basic negative ion conditions:* this optimized method used a separate dedicated C18 column (Waters UPLC BEH C18-2.1x100 mm, 1.7 µm). Methanol and water with 6.5mM ammonium bicarbonate at pH 8 were used to gradient elute basic extract.

*4) negative ionization:* after elution from a HILIC column (Waters UPLC BEH Amide 2.1x150 mm, 1.7 µm) using a gradient consisting of water and acetonitrile with 10mM ammonium formate, pH 10.8.

In parallel, three different types of controls were analyzed: (a) A technical replicate generated from a pool of human urine previously characterized by Metabolon, Inc., in detail; (b)

Process blanks extracted from water; and (c) a control to monitor instrument performance consisting of a cocktail of standards spiked into every analyzed sample.

All GCKD and control samples were randomly distributed across the platform run. A Waters ACQUITY UPLC and a Thermo Scientific Q-Exactive high resolution/accurate mass spectrometer interfaced with a heated electrospray ionization (HESI-II) source and Orbitrap mass analyzer operated at 35,000 mass resolution were used for all methods. Daily tuning and calibration for mass resolution and mass accuracy of instruments was conducted. The MS analysis alternated between data-dependent MSn scans and MS using dynamic exclusion. The scan range varied slighted between methods but covered 70-1,000 m/z.

The median relative standard deviation (RSD) for the spiked standards was computed to describe instrument variability (median RSD=5-7%; n=31 standards). To describe the overall process variability, the median RSD for all endogenous metabolites (i.e., non-instrument standards) present in all of the urine samples was calculated (median RSD=7-9%; n>1,000 metabolites). In Supplementary Data 1, RSDs for metabolites present in at least 90% of the pooled urine control samples are reported.

Identification of metabolites was based on an automated comparison of the ion features observed in the GCKD samples to a reference library of chemical standard entries including molecular weight (m/z), retention time, preferred adducts, and in-source fragments as well as associated MS spectra. Data were curated by visual inspection for quality control using software developed at Metabolon. Known chemical entities were identified by comparison to Metabolon's spectral library that contains >4,500 purified chemical standards. Commercially available purified standard compounds have been acquired to determine their detectable characteristics on the various platforms. Metabolites reported in this study as "known" conform to confidence level 1 (the highest confidence level of identification) of the Metabolomics Standards Initiative[3, 4], or are otherwise marked with an asterisk. In addition, >2,750 mass spectral entries of structurally unknown biochemicals that have been observed repeatedly in chromatography and mass spectrometry are available in the spectral library. The unknown molecules observed in the GCKD samples were also reported, with the potential for identification in once a matching purified standard is acquired or by classical structural analysis.

Metabolite levels were determined from peak quantification using area-under-the-curve (raw area counts) and normalized by the median value for each run-day to correct for variation due to inter-day tuning differences. Per metabolite, the median of levels from each run is therefore 1.0 and therefore comparable, while the variation between samples is preserved.

## References


1. Evans AM, DeHaven CD, Barrett T, Mitchell M, Milgram E. Integrated, nontargeted ultrahigh performance liquid chromatography/electrospray ionization tandem mass spectrometry platform for the identification and relative quantification of the small-molecule complement of biological systems. *Anal Chem* **81**, 6656-6667 (2009).

2. Evans A*, et al.* High resolution mass spectrometry improves data quantity and quality as compared to unit mass resolution mass spectrometry in high-throughput profiling metabolomics. *Metabolomics* **4**, 1 (2014).

3. Sumner LW*, et al.* Proposed minimum reporting standards for chemical analysis Chemical Analysis Working Group (CAWG) Metabolomics Standards Initiative (MSI). *Metabolomics* **3**, 211-221 (2007).

4. Schrimpe-Rutledge AC, Codreanu SG, Sherrod SD, McLean JA. Untargeted Metabolomics Strategies-Challenges and Emerging Directions. *J Am Soc Mass Spectrom* **27**, 1897-1905 (2016).
