# Supplementary material for: Rare genetic variants affecting urine metabolite levels link population variation to inborn errors of metabolism
Source: Nat Commun. 2021 Feb 11;12:964. doi: 10.1038/s41467-020-20877-8 (PMC7878905; doi:10.1038/s41467-020-20877-8)
Supplement: Supplementary file 3 — Description of Additional Supplementary Files [file 41467_2020_20877_MOESM3_ESM.pdf]

Description of Additional Supplementary Files

File Name: Supplementary Data 1

Description: Annotation of all evaluated metabolites.

File Name: Supplementary Data 2

Description: Study sample characteristics of GCKD participants

File Name: Supplementary Data 3

Description: Statistics of the qualifying variants contributing to significantly metabolite-associated genes.

File Name: Supplementary Data 4

Description: Summary statistics for genes associated with urine metabolites at genome-wide significance ($P<2.2E-6$) in at least one gene-based test (BURDEN/SKAT).

File Name: Supplementary Data 5

Description: Summary statistics for genes associated with a ratio of urine fatty acid or amino acid concentrations at suggestive significance ($P<2.2E-6$) in at least one gene-based test (BURDEN/SKAT) and with p-gain >537,140.

File Name: Supplementary Data 6

Description: Statistics of the qualifying variants contributing to significantly metabolite ratio-associated genes.

File Name: Supplementary Data 7

Description: Potential candidates for the identity of X-10457.

File Name: Supplementary Data 8

Description: Annotation of variants included in significantly associated genes.

File Name: Supplementary Data 9

Description: Evidence for implicated genes from OMIM and HMDB and agreement with metabolite changes observed in IEMs (metagene) and in earlier rare variant association studies.

File Name: Supplementary Data 10

Description: Genes with suggestive associations (P<6.3E-05) with binary traits in 50,000 whole exome sequences from the UK Biobank.

File Name: Supplementary Data 11

Description: Results from enrichment analysis (FDR<0.05) for the 30 significant genes across GO terms and KEGG pathways.

File Name: Supplementary Data 12

Description: Statistical power to detect rare variant associations across a range of effect sizes and minor alelle frequencies.
