# Supplementary material for: Rare genetic variants affecting urine metabolite levels link population variation to inborn errors of metabolism
Source: Nat Commun. 2021 Feb 11;12:964. doi: 10.1038/s41467-020-20877-8 (PMC7878905; doi:10.1038/s41467-020-20877-8)
Supplement: Supplementary file 5 — Reporting Summary [file 41467_2020_20877_MOESM5_ESM.pdf]

nature research

Corresponding author(s): Anna Köttgen

Last updated by author(s): Nov 24, 2020

Reporting Summary

Nature Research wishes to improve the reproducibility of the work that we publish. This form provides structure for consistency and transparency in reporting. For further information on Nature Research policies, see our Editorial Policies and the Editorial Policy Checklist.

Statistics

For all statistical analyses, confirm that the following items are present in the figure legend, table legend, main text, or Methods section.

n/a	Confirmed	
☐	☒	The exact sample size (*n*) for each experimental group/condition, given as a discrete number and unit of measurement
☒	☐	A statement on whether measurements were taken from distinct samples or whether the same sample was measured repeatedly
☐	☒	The statistical test(s) used AND whether they are one- or two-sided<br>*Only common tests should be described solely by name; describe more complex techniques in the Methods section.*
☐	☒	A description of all covariates tested
☐	☒	A description of any assumptions or corrections, such as tests of normality and adjustment for multiple comparisons
☐	☒	A full description of the statistical parameters including central tendency (e.g. means) or other basic estimates (e.g. regression coefficient) AND variation (e.g. standard deviation) or associated estimates of uncertainty (e.g. confidence intervals)
☐	☒	For null hypothesis testing, the test statistic (e.g. *F*, *t*, *r*) with confidence intervals, effect sizes, degrees of freedom and *P* value noted<br>*Give P values as exact values whenever suitable.*
☒	☐	For Bayesian analysis, information on the choice of priors and Markov chain Monte Carlo settings
☒	☐	For hierarchical and complex designs, identification of the appropriate level for tests and full reporting of outcomes
☒	☐	Estimates of effect sizes (e.g. Cohen's *d*, Pearson's *r*), indicating how they were calculated

Our web collection on statistics for biologists contains articles on many of the points above.

Software and code

Policy information about availability of computer code

Data collection	The conduct of the GCKD study is described in detail in the manuscript. All preparations and analyses of GCKD data was done at the Institute of Genetic Epidemiology, Medical Center - University of Freiburg, Freiburg (Germany). Metabolite measurements were performed as fee for service at Metabolon, Inc.
Data analysis	- Preprocessing of metabolites: R v3.5.1<br>- Software-tools for association analyses: in-house pipeline, R package SeqMeta v1.6.7<br>- Software-tool for postprocessing of RNA-seq data: Seurat v3.1.1<br>- Software-tool for statistical power: Quanto v1.2.4<br>- Software-tools for constraint based modeling and reconstruction analyses: COBRA Toolbox v3.0, Matlab v2018b, Ilog Cplex v 10.12, PSCM toolbox v1.0<br>- Miscellaneous: R v3.5.1

For manuscripts utilizing custom algorithms or software that are central to the research but not yet described in published literature, software must be made available to editors and reviewers. We strongly encourage code deposition in a community repository (e.g. GitHub). See the Nature Research guidelines for submitting code & software for further information.

Data

Policy information about availability of data

All manuscripts must include a data availability statement. This statement should provide the following information, where applicable:

- Accession codes, unique identifiers, or web links for publicly available datasets
- A list of figures that have associated raw data
- A description of any restrictions on data availability

All data are either publicly available or do not cover individual-level data sharing in the consent. Summary results with at least suggestive evidence of association are fully provided. Further details can be made available upon request.

Field-specific reporting

Please select the one below that is the best fit for your research. If you are not sure, read the appropriate sections before making your selection.

☒ Life sciences ☐ Behavioural & social sciences ☐ Ecological, evolutionary & environmental sciences

For a reference copy of the document with all sections, see nature.com/documents/nr-reporting-summary-flat.pdf

Life sciences study design

All studies must disclose on these points even when the disclosure is negative.

Sample size	We included all participants in the GCKD study with genotype, covariate and metabolite data (max. N=4864). Power calculations are shown as a Supplementary Table across a range of allele frequencies and effect sizes.
Data exclusions	Metabolites were excluded for high proportions of missingness (>80%). Samples were excluded if no genotypes, metabolites or covariates were available. This is clearly described in the methods.
Replication	Replication in a virtual whole-body, organ-resolved metabolic human. No other study with comparable metabolite measurements in urine and exome chip genotypes.
Randomization	Not relevant to this study because this is an observational study
Blinding	Not relevant to this study because this is an observational study

Reporting for specific materials, systems and methods

We require information from authors about some types of materials, experimental systems and methods used in many studies. Here, indicate whether each material, system or method listed is relevant to your study. If you are not sure if a list item applies to your research, read the appropriate section before selecting a response.

Materials & experimental systems

n/a	Involved in the study	
☒	☐	Antibodies
☒	☐	Eukaryotic cell lines
☒	☐	Palaeontology and archaeology
☒	☐	Animals and other organisms
☐	☒	Human research participants
☐	☒	Clinical data
☒	☐	Dual use research of concern

Methods

n/a	Involved in the study	
☒	☐	ChIP-seq
☒	☐	Flow cytometry
☒	☐	MRI-based neuroimaging

Human research participants

Policy information about studies involving human research participants

Population characteristics	Are described in Supplementary Data 2.
Recruitment	The GCKD study is an ongoing prospective observational cohort study of CKD patients. Between 2010 and 2012, 5,217 adult CKD patients under nephrological care provided written informed consent, were enrolled into the study and are currently followed for clinical endpoints over 10 years.
Ethics oversight	The GCKD Study was registered in the national registry for clinical studies (DRKS 00003971) and approved by local ethic committees. This information can be found in Eckardt et al, Nephrol Dial Transplant. 2012. PMID: 21862458.

Note that full information on the approval of the study protocol must also be provided in the manuscript.

Clinical data

Policy information about clinical studies

All manuscripts should comply with the ICMJEguidelines for publication of clinical research and a completedCONSORT checklist must be included with all submissions.

Clinical trial registration	This study is an observational study (DRKS 00003971).
Study protocol	The study protocol and design has been published in Eckardt et al, Nephrol Dial Transplant. 2012. PMID: 21862458.
Data collection	The GCKD study is an ongoing prospective observational cohort study of CKD patients. Between 2010 and 2012, 5,217 adult CKD patients under nephrological care provided written informed consent, were enrolled into the study and are currently followed for clinical endpoints over 10 years. For this project, urine specimens collected at baseline were selected for metabolite measurements.
Outcomes	Our primary outcomes were metabolites. Non-targeted MS analysis was performed at Metabolon, Inc. from urine samples collected at the study's baseline visit.
